# A Novel Scheme and Evaluations on a Long-Term and Continuous Biosensor Platform Integrated with a Dental Implant Fixture and Its Prosthetic Abutment

**DOI:** 10.3390/s151024961

**Published:** 2015-09-25

**Authors:** Yu-Jung Li, Chih-Cheng Lu

**Affiliations:** 1 Graduate Institute of Mechanical and Electrical Engineering, National Taipei University of Technology, Taipei 10608, Taiwan; E-Mail: richard513.tw@yahoo.com.tw; 2 Department of Mechanical Engineering, National Taipei University of Technology, Taipei 10608, Taiwan

**Keywords:** dental implant system, intra-oral biosensor platform, glucose oxidase (GOD), blood sugar level, Diabetes Mellitus (DM)

## Abstract

A miniature intra-oral dental implant system including a built-in biosensor device is proposed in this article. The dental implant system, or platform, is replaced over maxilla and allows relatively non-invasive procedures for a novel biosensing scheme for human blood analysis. Due to placement of the implant fixture, periodontal ligaments and the pulp structure, which are regarded as the main origin of pain, are thus removed, and long-term, continuous blood analysis and management through maxillary bone marrow becomes achievable through the dental implant platform. The new pathway of biological sensing is for the first time presented to realize an accurate and painless approach without injections. The dental implant system mainly consists of an implant fixture and a prosthetic abutment, a biosensor module, a bluetooth 4.0 wireless module and a dc button cell battery. The electrochemical biosensor possesses three electrodes, including working, reference and counter ones, which are arranged to pass through the titanium implant fixture below the biosensor module. The electrodes are exposed to the blood pool inside the maxillary bone marrow and perform oxidation/reduction reactions with the coating of biosensing enzyme. To prove the proposed platform, the immobilization process of glucose oxidase (GOD) enzyme and *in vitro* detections of glucose levels are successfully carried out, and proven sensitivity, linearity and repeatability of the glucose biosensor system are obtained. Moreover, a preliminary canine animal model adopting the new pathway shows significant consistency with the traditional method through dermal pricks for blood sugar detection. Despite the prospective results, further challenges in engineering implementation and clinical practices are addressed and discussed. In brief, the novel biosensing pathway and intra-oral biosensor platform may increasingly reveal their promising value and feasibilities in current bio-medical analysis, diagnosis, drug release and even healthcare technologies.

## 1. Introduction

Along with the increased average life expectancy, diagnostic management for chronic and critical diseases is becoming more important in the early twenty-first century. Clinical or home healthcare analysis from online monitoring data is thus considered indispensable for the further diagnosis and other medical procedures. In addition, the concept of long-term and continuous bio-supervision or bio-monitoring turns out to be more essential in modern medicine for both healthcare and diagnostic procedures than ever. These monitoring approaches are classified into two categories: invasive and non-invasive procedures. For instance, blood pressure (BP), respiration rate (RR), and heart rate (HR) measurements are categorized as non-invasive procedures, while most of blood analysis procedures are still considered invasive due to inevitable dermal or mucosal irritations. Blood analysis offers plenty of bio-chemistry data that would have great influence on differential diagnosis. For example, the blood urea nitrogen (BUN) and serum creatinine (Cr) value in renal function evaluation, The AST/ALT ratio (aspartate transaminase–alanine transaminase ratio or aspartate aminotransferase–alanine aminotransferase ratio) corresponding to the hepatic cell damage, and the cardiac marker such as Myoglobin, creatine kinase (CK), Cardiac Troponin I in acute myocardiac infraction (AMI) diagnosis, are all proven to be significant markers in diagnosis [1]. On the other hand, due to its invasive attribution, frequent and continuous blood monitoring is highly limited as dermal destruction process is pain-arising and only performed in critical or emergent patients with indwelling needles or catheters in vein and artery [2]. A well-known example of metabolic disorder is Diabetes Mellitus (DM), leading to relative renal and cardiovascular diseases in hundreds of millions of people. Due to high blood sugar value and relative ischemic status, patients will also remain in a high risk of infection. In the later stage, DM patients may be regularly arranged for four invasive blood sugar detection and subcutaneous injections of insulin to control blood sugar levels each day [3]. Such detection procedures may bring mental stress and physical discomfort at the same time. Currently, the most popular method for blood sugar detection is the dermal or mucosal irritations via an invasive penetration for blood sampling, and the biosensor operates mainly through glucose oxidation reaction using glucose oxidase (GOD) catalysis [4]. Despite the stress and irritations, however, recurrent blood analysis and monitoring is a routine practice in most medical procedures for patients in hospitals, especially for the elderly with chronic or other critical diseases. Recently, though there have been several non-invasive methods or techniques such as subcutaneous glucose monitoring developed to measure the bio-medical parameters, these methods are still immature and unable to be effective diagnostic tools for clinic applications [5]. As a result, it is very urgent and vital to find an alternative pathway to complete bio-medical analysis, and, in the meantime, minimize the discomfort during these invasive medical procedures so as to provide the patients with improved quality of life.

Dental implants have been widely accepted as an advanced dental procedure for tooth reconstructions since Dr. Brånemark first introduced the concept of osseointegration in dental implant in the 1970s [6]. Today this kind of surgical techniques in dental implantology along with the cylindrical pure titanium implant body develops into a standard medical procedure and broadly applied in clinical practice [7]. The implant surgery is mainly performed under local anesthesia and divided into two stages. In the first stage, the jawbone is exposed after flap design, and the titanium implant fixture is carefully placed into the created bony socket with initial stability. After a period of 3~6 months for mature bone healing, the cortical bone structure will closely contact the implant surface, which is referred as osseointegration. In the second stage, the surgery can be arranged to expose beneath implant fixture [8], and then the prosthetic abutment is jointed above the fixture. Finally, under proper impression and other relative dental procedures, the prosthetic crown or bridge is delivered over the abutment. In the whole dental procedure, the cylinder-type fixture is about 8~15 mm in length and 4~5 mm in diameter depending on the jawbone morphology; while the prosthetic crown in bilateral posterior molar area is in the average dimensions of 15 mm × 10 mm [9].

From the anatomic point of view, the origins of the tooth sensation are mainly from the pulp structures and the periodontal ligaments (PDL) [10]. With the artificial implant fixtures placement into jawbones, these paths of major painful sensation are both removed and this fact thus proposes a potential method for relatively painless blood analysis and monitoring techniques notwithstanding the risk of inflammation or peri-implantitis. Further on the anatomic structure of jawbones, due to the structural difference between maxilla (the upper jawbone) and mandible (the lower jawbone), the spongy and more chancellors bone structure on maxilla can offer the rich blood supply for diagnostic analysis and assure the biosensor’s electrodes to be inserted to the blood pool [11]. In addition, its chancellors structure may work as the cushion to lower down the discomfort from the surrounded pressure receptors. Furthermore, the intra-bony blood pool may also offer another prospective application in continuous and painless drug release [12].

**Figure 1:**
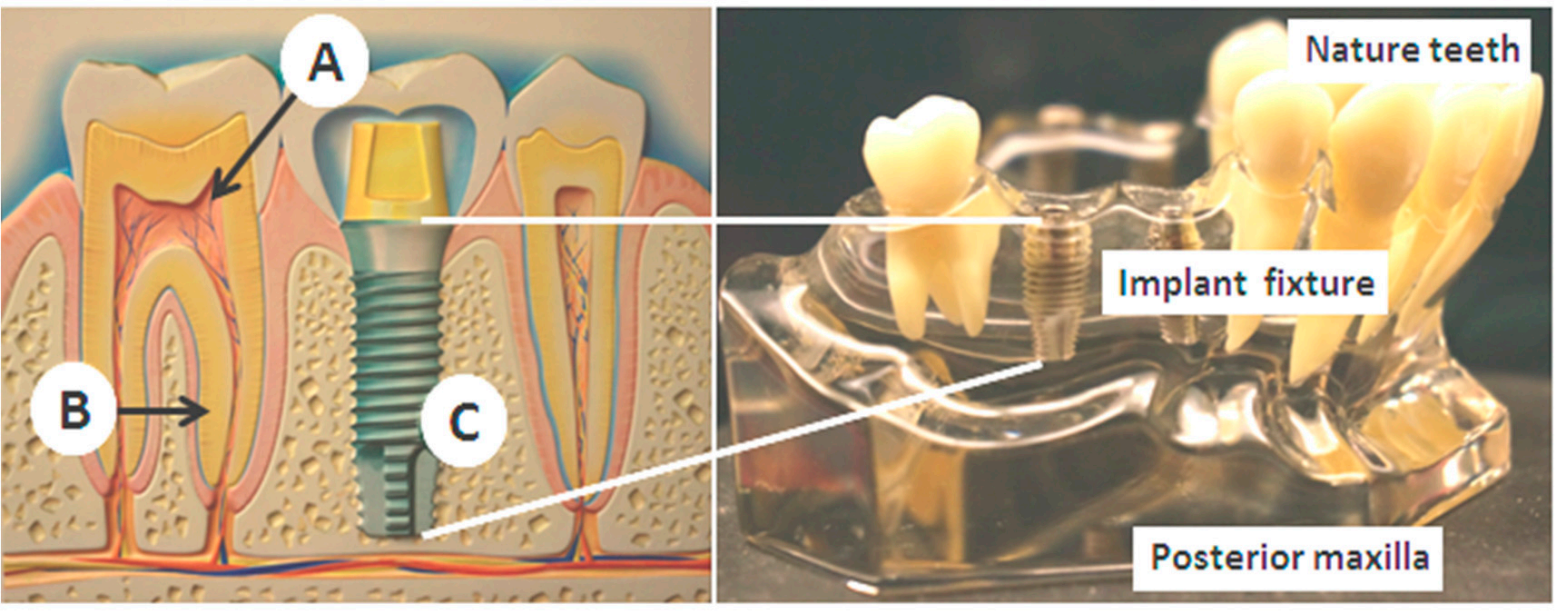
The intra-oral environment over maxilla and the tooth structures. The pain origins are mainly from the pulp structures (A), and the periodontal ligament (B). After artificial implant restoration (C), they are both removed.

Inspired by the above anatomic facts and currently mature intra-oral implant techniques, we herein present for the first time a novel concept of miniature bio-sensing platform embedded into the maxillary implant fixture for a long-term, continuous and painless blood analysis technique in this article. As shown in Figure 1, after removal of the pulp structure (A) and the periodontal ligament (B), the artificial titanium implant has a lack of pain receptors and will lead to painless status (C). The principal design of the bio-medical sensing platform as well as GOD enzyme coating and voltammetric calibration procedures of the biosensors are first described. Next, followed are the implementations of the transducing circuit for blood sugar detection and the wireless transmission measurement of the bluetooth 4.0 module. In addition to these engineering verifications, a proven canine experiment demonstrates efficient insulin release and successful detection of blood sugar levels. These preliminarily positive results provide us commercial and technical feasibilities for a new biosensing way in the near future. Finally, concluding remarks and the outlook for the proposed bio-medical platform are presented. More improvement works on the novel platform are currently under way in our laboratory.

## 2. Design, Methods and Experiments

### 2.1. Design Structure of the Implant-Based Biosensor

**Figure 2:**
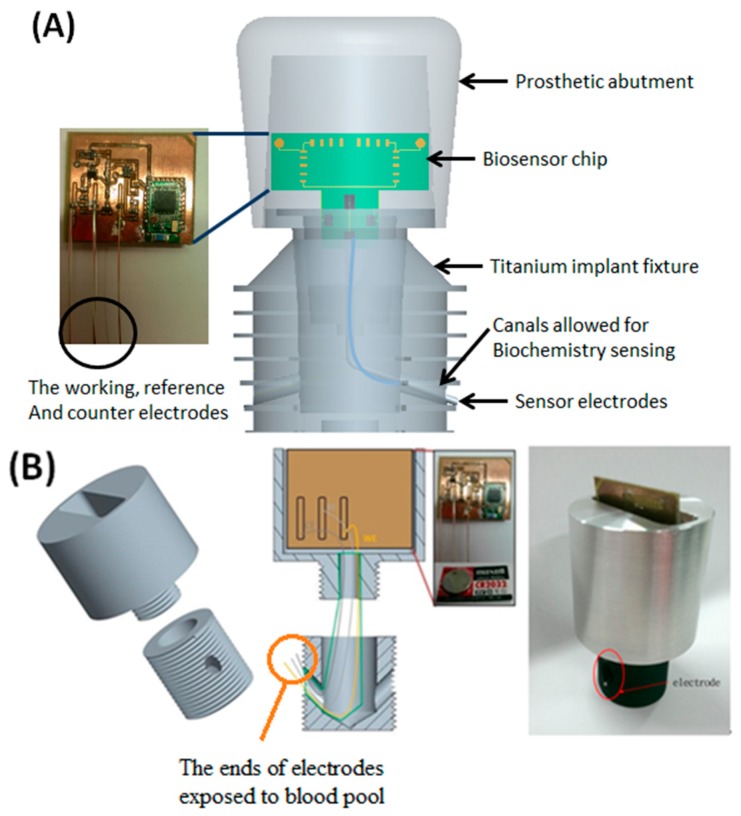
The design of the intra-oral implant-supported biosensor over maxilla. (**A**) marks each components of the module, including a set of the electrodes; and (**B**) shows the prototype biosensor with electrodes exposed to outside blood pool as denoted.

The proposed platform is basically designed for various sensing arrangements for blood monitoring as long as they are small enough for installation. Figure 2 illustrates the unique design of the intra-oral implant-supported system. The implant platform mainly consists of a prosthetic abutment and a pure titanium implant fixture, which is directly connected to the maxillary bone structure. This prototype is 4.0 cm × 2.5 cm in size, which is about twice the target dimensions (2 cm × 1.2 cm) for preliminary engineering evaluation. The preliminary design of the electrochemical biosensor contains a set of tri-electrodes, a transducing circuit board along with button cells as power supply, and a wireless bluetooth 4.0 module to transfer the measured data to an external recipient server, such as cell phones, personal computers or other machines with micro control units (MCUs). All of the above biosensor components are inserted into the prosthetic abutment where the intra-oral electrochemical biosensor can prevent the occlusal loading of teeth. Besides, two individual canals or channels in the implant fixture 1 mm in diameter allow three sensing electrodes to pass through the surface of the implant fixture and immerse in the blood pool inside the maxillary bone marrow. A set of tri-electrodes includes working, reference and counter electrodes, which are isolated from each other to circumvent short circuit and current leakage.

The implant fixture and prosthetic abutment with the tri-electrodes and channels is required to place into the patient’s maxilla in the beginning. Three sensing electrodes in the abutment will extend out of the fixture afterward through the channels to expose themselves to the maxillary blood pool outside and then directly contact the surrounding bone marrow. Finally, in the post-sensing phase, wireless data transmission is very important in such biomedical sensor application. Due to the properties of low energy consumption, a higher transmission rate and a wider range, the bluetooth 4.0 module is adopted as our wireless communication unit [13], as it is able to obtain the complete set of data regarding analyte concentrations in blood measured from the biomedical sensing system.

### 2.2. GOD Immobilization Procedure and Glucose Calibration

Diabetes Mellitus is a well-known metabolic disorder, which will lead to relative renal and cardiovascular diseases, and patients with such a disease will usually lead to regularly invasive procedures with both blood sugar monitoring and insulin delivery eventually, which can avoid relative renal failure and other cardiovascular complications. Thus, proper and regular management of blood sugar levels is rather important for DM control. By using the usual finger pricking approach, one of the common methods for monitoring the blood glucose levels is to apply glucose oxidation reactions with glucose oxidase (GOD) catalysis, and the chemical equations are as follows [4,14,15,16]:

$$\text{β}-\text{D}-\text{Glucose }+{\text{ O}}_{2}\;+{\text{ H}}_{2}\text{O}\overset{\text{ glucose oxidase }}{\to }\text{D}-\text{Gluconic acid }+{\text{ H}}_{2}{\text{O}}_{2}$$

Basically, GOD is a dimer structural protein with molecular weight equals to 126,000. Thus it is important to immobilize GOD onto the electrode surface with proper chemically binding modifications, and these immobilization techniques may be different from materials or protocols in laboratories. The materials and protocols employed in this work are as follows:

Materials:

Pure gold electrode with 0.5 mm in diameter, Crosslinker agent as Stock 1, two kinds of coupling agent as Stocks 2 and 3 (store at −20 °C), GOD enzyme as Stock 4 (store at 4 °C), 100% Ethanol, and Phosphate buffer saline (PBS).

Protocol:

(1) Gold electrode washing in distilled water and 100% ethanol.
(2) Gold electrode immersing Stock 1 in the ethanol solution for 24 h.
(3) Gold electrode washing in distilled water and 100% ethanol again.
(4) Coupling agent preparation by mixing 10 μL each of Stocks 2 and 3.
(5) Incubation for 1 hour after adding the coupling agent onto the gold electrode surface.
(6) After PBS washing, add 20 μL of GOD enzyme solution as Stock 4, and then incubate at 4 °C for 2 h.
(7) Then the enzyme-modified electrode is ready for cyclic voltammetric operation.

Finally, the glucose solutions in our experiments are prepared in 50 mg/dL, 100 mg/dL, 200 mg/dL, 300 mg/dL, 400 mg/dL, *etc*. to fit the measurement range for clinical applications of the biosensor system.

### 2.3. Design and Verification of the Transducing Circuit

**Figure 3:**
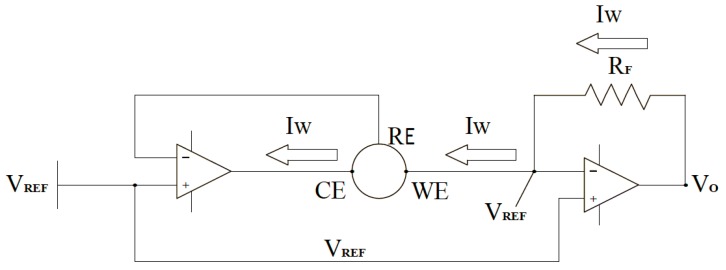
The brief circuit diagram in the glucose sensing device with two amplifiers, the working (WE), counter (CE), and reference (RE) electrodes for glucose sensing.

Figure 3 shows a brief schematic of the electrochemical sensor measurement circuit in our experiments. Electrochemical sensors work by allowing fluid to diffuse into the sensor and interacting with the working electrode (WE). The sensor reference electrode (RE) provides feedback to maintain a constant potential with the WE terminal by varying the voltage at the counter electrode (CE). The direction of the current at the WE terminal depends on whether the reaction occurring is oxidation or reduction. In the case of carbon monoxide, oxidation takes place; therefore, the current flows into the working electrode, which requires the counter electrode to be at a negative voltage (typically 300 mV to 400 mV) with respect to the working electrode. In our experiment, the current into the WE terminal is less than 100 nA per ppm of the liquid concentration. Thus, converting this current into an output voltage requires a transimpedance amplifier with a very low input bias current. The amplifier (AD8500 Operational Amplifier Analog Devices, Cambridge, MA, United States) has CMOS inputs with maximal input bias current of 1 pA at room temperature, making this amplifier very appropriate for the application. Furthermore, the 2.5 V power source (LM385) establishes the pseudo-ground reference for the circuit, which allows for single-supply operation while consuming very little quiescent current.

Amplifier sinks enough current from the CE terminal to maintain the zero potential voltage between the WE and RE terminals of the sensor. The RE terminal is connected to the inverting input, which stops any current flow passing through it. This results in all the current passing through the WE terminal, which leads the current to show a linear response to the contact glucose concentration. Thus, transimpedance amplifier converts the current through the working electrode into the corresponding voltage proportional to the glucose concentration in liquid.

### 2.4. Design and Manipulation of Transducing Circuit

The output voltage of the transimpedance amplifier is described below:

(1) $$V_{0}=0.7\text{V}+I_{WE}\times R_{F}$$

where *I_WE_* is the current into the WE terminal, and *R_F_* is the transimpedance feedback resistor. The maximum response of the sensor is 58.8 nA/(mg/dL), and its maximum input range is 400 mg/dL of carbon monoxide. This results in a maximum output current of 23.5 μA, and a maximum output voltage determined by the transimpedance resistor, as shown in Equations (2) and (3):

(2) $$V_{0}=0.7\text{V}+400\text{mg/dL}\times 58.8\frac{\text{nA}}{\text{mg/dL}}\times R_{F}$$

(3) $$V_{0}=0.7\text{V}+23.5\text{μA}\times R_{F}$$

By the circuit with a 3 V power supply will result in a usable range of 0.7~3 V at the output of the transimpedance amplifier. By selecting a 100 kΩ resistor for the transimpedance feedback resistor, we can get a maximal output voltage of 3 V, which allows for approximately 8% of over range capability. The resistor (*R**_F_***) keeps the noise gain at a reasonable level. Selecting the value of this resistor is a compromise between the magnitude of the noise gain and the error of sensor settling time when exposed to high glucose concentrations. For this example, *R**_F_*** = 330 Ω, which results in a noise gain (NG) of 304, as shown in Equation (4):

(4) $$NG=1+\frac{100\text{kΩ}(R_{F})}{330\text{Ω}}≅304$$

The input noise of the transimpedance amplifier appears at the output amplified by the noise gain. For this circuit, we are only interested in the low frequency noise because that from the sensor operation maintains at low level. In Equation (5), the AD8500 amplifier has an input voltage noise of 6 μV_p-p_ from 0.1 to 10 Hz in frequency, which results in the output voltage of 1.82 mV_p-p_.

(5) $$V_{out}=6\text{μV}\times NG=1.82{\text{mV}}_{\text{p-p}}$$

It is hard to filter out the noise because it appears at very low frequency. However, the sensor response corresponding to the noise is also at the very low frequency level. Thus we choose a low-pass filter (resistor and capacitance) for very low frequency bandwidth and it is with a cutoff frequency of 0.16 Hz for the noise. Here it should be noted that, compared with the 30 s of response time for the sensor, the effect on the sensor response time is negligible even with such a low frequency filter.

Finally, one of the important characteristics among these electrochemical sensors should be their long response time compared with other types of sensors. Basically, after system power on, the biosensor may take 1~2 min to settle down for its final output value. When exposed to a specific concentration of the target liquid, it may require from 25 to 30 s for the sensor output to reach 90% of its final value. Thus the response time, on average, for the biosensor is estimated as 2 min. If the voltage between the RE and WE terminals has an abrupt change in magnitude, it may take several minutes for the sensor current output to settle.

### 2.5. The Bluetooth Module and Data-Logging Interface

**Figure 4:**
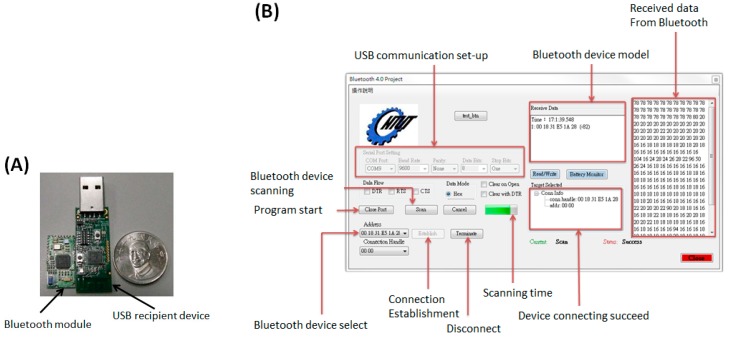
(**A**) A bluetooth 4.0 module and its USB recipient device; (**B**) The interface of the bluetooth control panel completed by Visual C# and its function description.

The bluetooth 4.0 module shown in Figure 4A is Model CC2540 with 2.4 GHz bluetooth low energy system-on-chip produced by Texas Instruments Corp. Major specifications of the wireless module includes: a 128/512 Kb flash memory and 8 Kb SRAM inside, a 21 universal I/O interface, 2 USB 2.0 ports, an advanced 8 channel 8–12 bit analog to digital (AD) converter, low energy consumption and maximum +97 dB link budge [13]. The bluetooth 4.0 module is obviously smaller than the USB recipient device, as shown on the right hand side. The control program for data logging/transmission was implemented by Visual C# in Microsoft Visual Studio 2010. The complete interface shown in Figure 4B can record and display the transmitted digital data for glucose sensing procedures from the bluetooth 4.0 module.

### 2.6. Animal Model Experiment

The one-year-old canine’s bilateral maxillary first premolar was extracted and prepared for dental implantation. Two of the 6 × 5 mm Bicon^©^ titanium fixtures were implanted over bilateral maxilla on premolar area under barbiturate anesthesia. After 1 month of healing, the fixtures were exposed under local anesthesia, and the drug delivery syringe (DDS) was placed on maxillary bone marrow for insulin release on the right hand side. The integrated biosensor fixture will be placed on the implant fixture on the left hand side to detect the change of blood sugar levels.

**Figure 5:**
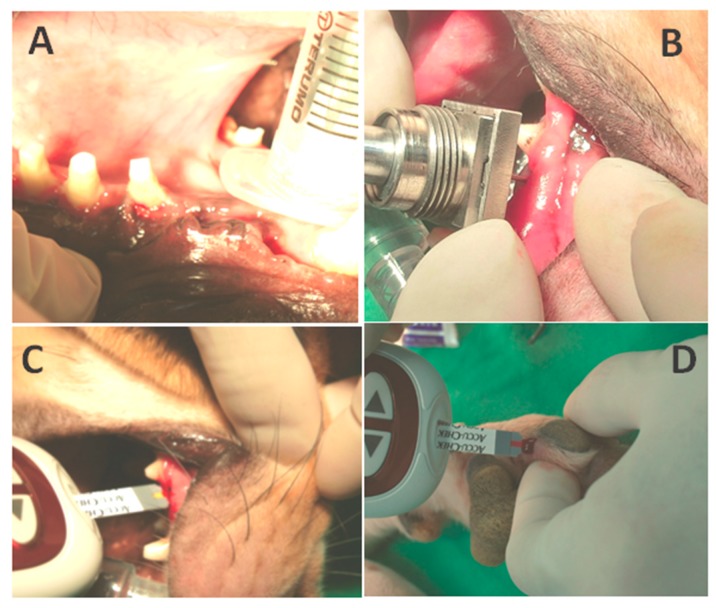
The canine model for blood sugar monitoring. (**A**) The wound exposure after 1 month for further implant placement; (**B**) The DDS and biosensor module for insulin releasing and blood sugar monitoring through the maxillary pathway; (**C**) Blood sugar calibration via the maxillary pathway by ACCU-CHEK® blood sugar detector; (**D**) Blood sugar detection via the anterior limb.

Initially, before the insulin releasing procedure, the blood sugar level of the canine was measured twice to show the canine’s baseline value. Five units of the Neutral Protamine Hagedorn (NPH), also known as rapid onset insulin, was then loaded on the DDS and released into the maxilla. After insulin release, the blood sugar values were detected every 5 min by both the maxillary implant biosensor and an ACCU-CHEK^®^ Performa blood sugar detector (Roche company, Basel, Switzerland), as shown in Figure 5, representing the preliminary canine model experiment. The ACCU-CHEK^®^ detector is responsible for the control purpose of the subcutaneous blood sugar levels. Figure 5A shows the surgical wound after first titanium implant placement surgery, Figure 5B represents the proposed biosensor platform and the DDS to monitor blood sugar and deliver insulin, respectively. Finally, Figure 5C,D represents the blood glucose calibration operations over the canine’s maxilla and anterior limb by ACCU-CHEK^®^ blood sugar detector.

## 3. Results

The main results in our experiments can be divided into three parts. First of all, we clarify the relationship between logged bluetooth data and the output voltage of the biosensor, which corresponds to the resistance caused by different glucose concentrations. Next, we evaluate the quality of the GOD immobilization by cyclic voltammetry techniques, and investigate the proportional relationships between the glucose concentrations and the reactive currents. We also monitored solutions with different glucose concentration by the biosensor module, and analyzed bluetooth data. Finally, we perform the canine animal model for blood sugar monitoring to evaluate validity of the new detection pathway and repeatability of the biosensor module.

### 3.1. Resistance Modulation and Bluetooth Transmission Verification

**Figure 6:**
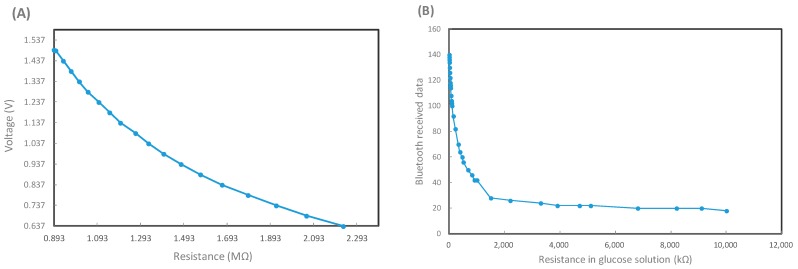
The calibration process for the glucose sensor and bluetooth 4.0 module. (**A**) The relationship between transducing voltage *vs.* input resistance in the circuit, which may simulate different glucose concentrations in solutions; (**B**) The logged bluetooth signal *vs.* input resistance standing for different glucose solutions in the transducing circuit.

**Figure 7:**
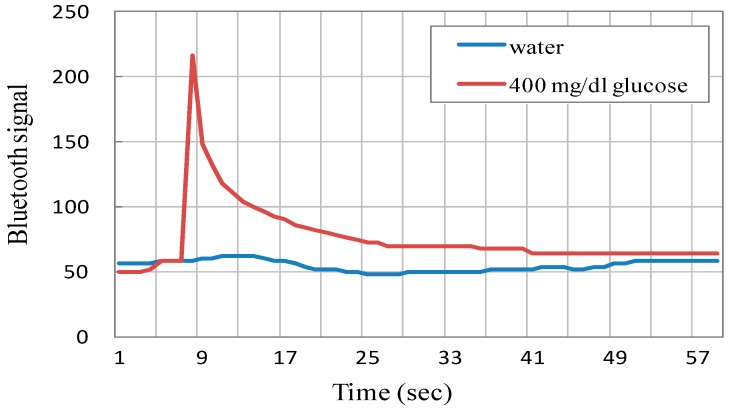
The temporal change of logged bluetooth signal data sets with respect to distilled water and a 400 mg/dL glucose solution.

Bluetooth module is responsible for signaling transfer in the biosensor. Thus calibrations at first are important. During cyclic voltammetry (CV) sweeping, the applied voltage is around −5 to +5 V, and the resistance changes may correspond to various glucose concentrations. Figure 6A shows voltage changes due to various input resistances over the working electrode in the biosensor circuit, while Figure 6B represents the relationship between the input resistances and bluetooth signal data. Both relationship patterns may not totally follow a linear response to each other, but they move linearly toward each other at intervals. This will allow for the evaluation of glucose concentrations. It should be noted that since the biosensor demonstrates a period of time delay in measurement, each of the transmitted measurement is required to last for one minute to confirm its validity, as shown in Figure 7. The bluetooth signal data from distilled water as a negative control is around 50 bluetooth units and can be regarded as background noise. On the other hand, e.g., the signal triggered from a 400 mg/dL glucose solution, exhibits a clear peak within the first 10 s, and gradually degrades to the background baseline beyond about 50 s. Therefore, the peak value will represent the bluetooth signal from a specific glucose concentration in solution.

### 3.2. Cyclic Voltammetry and Calibration of the Glucose Biosensor System

**Figure 8:**
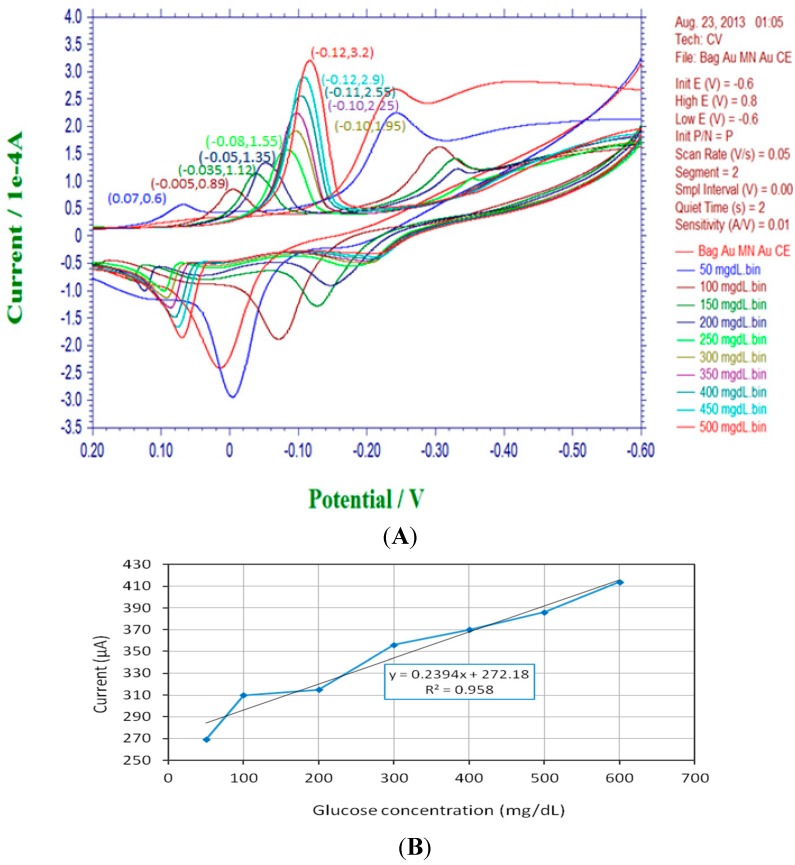
Cyclic voltammograms and calibration plot of the intra-oral biosensor module. (**A**) The cyclic voltammetric sweep shows the electrochemical relationships among current and applied potential with respect to different glucose concentrations (0~500 mg/dL); (**B**) The sensitivity expression and calibration plot of the amperometric biosensor.

With a proper GOD immobilization procedure, the current through the working electrode shows highly positive correlations to glucose concentrations in cyclic voltammetric analysis. Figure 8A shows the typical cyclic voltammograms illustrating the responses among current, applied potential and specific solutions with different glucose concentrations due to electrochemical interactions. After rearrangement of the peak values of electrode currents, as shown in Figure 8B, it is observed that the sensor demonstrates approximately linear behavior between the glucose concentration and sensor current. The slope of the linearly fitted curve, equivalent to sensitivity of the basic biosensor, is found to be about 240 nA/(mg/dL). Hence, it can be inferred that a 4 mg/dL increase in the glucose concentration is able to boost the current higher for about 1 μA. The CV sweeping experiment is very helpful to evaluate the GOD immobilization effect over working electrodes and offer the calibration basis for further wireless signal translations in blood sugar monitoring.

### 3.3. Repeatibility Test of the Glucose Biosensor Platform

**Figure 9:**
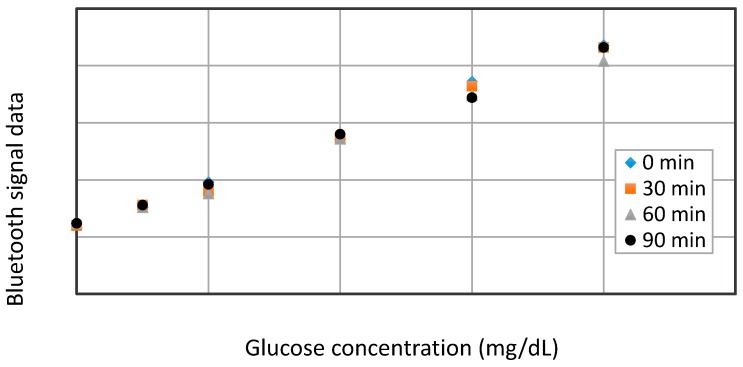
The repeatability test results of the glucose biosensor.

To ensure the constant accuracy and repeatability of the biosensing system, it is vital to operate the biosensor at baseline point, 50, 100, 200, 300, and 400 mg/dL in glucose solutions with an interval of 30 min, as depicted in Figure 9. According to the transmitted bluetooth peak signals, the biosensor exhibits better repeatability at lower concentrations under 200 mg/dL than that at higher concentrations, and the errors of detection among different time-interval measurements are verified to remain less than 10%, even at a higher concentration of 400 mg/dL. Based on our completed experiments, it is estimated that the repeatability of the biosensor may last for about 8~10 h, until the immobilized GOD enzyme degrades in the end.

### 3.4. Insulin Release and Blood Sugar Detection over Canine Model

**Figure 10:**
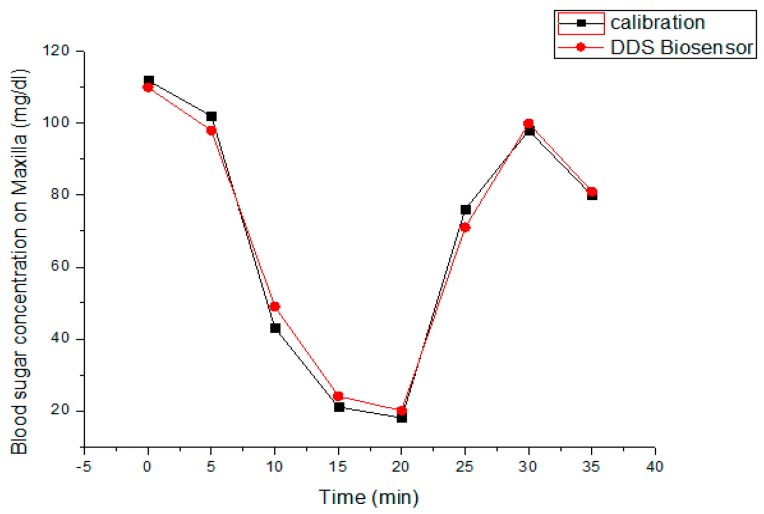
The temporal variation of blood sugar levels measured by the proposed intra-oral implant system and ACCU-CHEK^®^ blood sugar detector.

After primary osteointegration for one month, the canine received five units of NPH loading throughout the DDS system over maxilla. The initial blood sugar level detected by our biosensor over canine’s maxillary implant was 112 mg/dL, and, for comparison, the control value measured by an ACCU-CHEK^®^ blood sugar detector was about 118 mg/dL. The blood sugar detection was performed every 5 min subsequently after NPH loading. Figure 10 illustrates a brief temporal change of blood sugar levels with our biosensing implant system and an ACCU-CHEK^®^ blood sugar detector. It is clearly shown that the blood glucose levels, after five minutes of initial NPH loading, was rapidly decreased in both biosensors due to the NPH loading effect. However, the glucose levels then rebounded from 18 mg/dL, and more promptly approached to the stable level about 80 mg/dL after about half an hour. Here the rebounding effect of blood sugar level for the canine may be justified as the physiological feedback due to the reactive glucogen secretion after a rapid decrease of blood sugar level.

## 4. Discussions and Outlook

The purpose of the intra-oral implant-based biosensor platform is for long-term and continuous blood analysis and monitoring, and the resultant advantages of this sensor platform are as follows:

(1) Compared to other implanted biosensors, the proposed sensor is fixed by surrounding bone tissue, which will reduce the risk of component loosening and internal bleeding problems.
(2) The use of the implant fixture and prosthetic abutment will result in several benefits including biosensor module replacement without any additional surgery, regular sterilization at dental clinic in a non-invasive way, and the easy substitution of the system power supply with a dc button cell battery.
(3) By mature dental implant procedures, further maintenance of the biosensing system may go along under apical X-ray films with minimized dosage. Other periodontal curettage may also be arranged to maintain the sensor in a workable condition if needed.

In this preliminary evaluation, we design the intra-oral biosensor prototype, which is about twice the size of a comparable unit currently used in practice for clinical applications, to reduce the existing difficulties such as specific design and manufacture for compact components, GOD enzyme immobilization on fine electrodes, and system assembly. On cyclic voltammetry and its resultant bluetooth signals, the current shows reasonable linearity with the glucose concentration. Thus every 2 mg/dL glucose concentration will increase the current to about 1 μA and correspond to 0.8 reading unit of bluetooth signal. However, the ideal linearity is evaluated in pure glucose solution *in vitro*, and the noise may become more significant when it applies to real blood due to its complicated components. Besides, as shown in Figure 9, the GOD enzyme exhibits good repeatability and accuracy in laboratory in pure glucose solutions. In spite of these characteristics, it is noted that GOD enzyme tends to degrade within 48 h and result in lower signal response with time. This issue thus becomes challenging if the biosensing platform is expected to be employed as a long-term application, e.g., valid for 30 days. Therefore, further improvement of GOD coating or the use of temporal calibration are needed for clinical practice. Moreover, the 5 V button cell battery may last for about one month under the operation frequency of continuous detection every 5 min, which satisfactorily meets the requirements of the system applications.

For further clinical applications, one should carefully deal with the dental procedures under infection control and other safety problems. Fortunately, mature dental implant procedures have provided hints of long-term care for the intra-oral biosensor platform. At first, a tight connection to the fixture and abutment will isolate the inner bone marrow structure from outside contaminations. This tight connection will also prevent significant blood loss during continuous blood monitoring. Further, regular dental appointments with the replacement of battery and biosensor module after proper H_2_O_2_ irrigation will also cut down the risk of infection. Moreover, the blood-rich marrow structure will also minimize the probability of thrombosis compared to other intra-venous analyzing procedures. To sum up, the intra-oral biosensor platform is required to fulfill the following facts or requirements:

(1) Prevention from the occlusal loading forces to circumvent unexpected saliva micro-leakage, which may result in further contaminations, sensor damage, and chronic marrow infections.
(2) GOD or other enzyme coating improvements maybe based on proper polymerization to maintain continuous sensor operation at least for one month.
(3) The power supply scheme for the platform in both safety control and long-term reliability. In addition to the miniature battery solution, the emerging wireless charging technique may also be taken into consideration in the near future.
(4) Further minimization of the biosensor module is extremely necessary as the smaller size it will be, the less discomfort and side effect it may cause.
(5) More *in vivo* animal experiments are expected, as long-term histology evaluations will result in improvement of the safety requirements.

On limitations of the biosensor, it is noted that the proposed platform can be mainly suitable for patients with the requirements of regular invasive procedures. For example, type I diabetes mellitus (DM) patients need to receive the punches to monitor blood sugar four times a day, and patients receiving hemodialysis may face a similar situation. The proposed device may minimize patients’ sufferings when they face frequently invasive arrangements. For healthy and fully dentulous people, it seems unnecessary to place this class of bio-sensing device unless they are willing to do so for the reason of future health care. In addition, the intra-oral implant-based biosensor also includes the following restrictions:

(1) The biosensor is specifically designed for some patient groups with the demands for long-term and regular blood monitoring, especially for the elderly with chronic and/or critical diseases. However, elderly people might mostly have edentulous ridge.
(2) The fabricated cost of this intra-oral implanted device would be much more expensive compared to other biosensing devices or approaches.
(3) With the more complicated biosensor, the risks of infection and peri-implant issues may increase, and the user thus needs more dental appointments for maintenance procedure, which may not be an economical process in some countries.

Finally, this intra-oral implant-supported analysis platform can be regarded as a kind of novel biosensor that can directly achieve accurate blood analyzing functions with minimal painful sensation. Without the need of dermal destruction or injection, various classes of developed electrochemical biosensors or those in development can be integrated with the proposed system. More importantly, this new approach could be exceedingly superior to the non-invasive blood analysis techniques such as optic or transdermal detection methods, particularly in sensitivity and accuracy. In this work, we select blood sugar as the analyte because it is the most often seen in daily life. Other feasible analytes in blood analysis items with this biosensor system may also be considered and customize in specific medical procedures. For example, continuous monitoring of cardiovascular markers toward the patients with high risk factors may decrease stroke possibilities or avoid delayed administrations. As modern medicine evolves, geriatrics has become much more important than ever before, so that the quality of life should also be taken into account. Since this work is a preliminary study, more advanced module designs, animal model tests, and clinical practice assessment are needed for system advances and reliability assessment.

## 5. Conclusions

We report a new class of an intra-oral biosensor platform embedded in a dental implant fixture and its prosthetic abutment, which may meet the needs of long-term, continuous and accurate blood analysis through a relatively painless pathway. The newly proposed method of biological sensing is presented for the first time to realize a non-invasive blood monitoring way in a painless manner without injections. As a novel and miniature biosensing platform, in addition to blood sugar levels, biochemistry data such as CK, Troponin I, and so on can be selected and developed by the use of replaceable modules. In this study, a number of works including design and assembly of the implant-based biosensor platform, implementation and communication of the transducing circuit and wireless bluetooth 4.0 module, biochemical synthesis of GOD enzyme and immobilization verification, performance tests of the biosensor platform, and a preliminary canine model experiment on blood sugar level detection are successfully carried out. In summary, these experimental results indicate very high feasibility of success for the proposed biosensor system. Nevertheless, the integrated biosensing platform needs constant efforts or improvements in system miniaturization, the infection control routine, advanced or smart control on automatic analysis, enhanced reliability, increased lifetime of the biosensor, and even business model assessment. More importantly, the realization of this new biosensing platform may have a significant influence on current medical diagnosis and therapy, especially for chronic diseases and elderly patients with critical diseases.
